# Recent trends in stream macroinvertebrates: warm-adapted and pesticide-tolerant taxa increase in richness

**DOI:** 10.1098/rsbl.2021.0513

**Published:** 2022-03-23

**Authors:** Friederike Gebert, Martin K. Obrist, Rosi Siber, Florian Altermatt, Kurt Bollmann, Nele Schuwirth

**Affiliations:** ^1^ Swiss Federal Institute for Forest, Snow and Landscape Research WSL, Zürcherstrasse 111, 8903 Birmensdorf, Switzerland; ^2^ Eawag: Swiss Federal Institute of Aquatic Science and Technology, Überlandstrasse 133, 8600 Dübendorf, Switzerland; ^3^ ETH Zurich, Institute of Biogeochemistry and Pollutant Dynamics, Universitätstrasse 16, 8092 Zürich, Switzerland; ^4^ Department of Evolutionary Biology and Environmental Studies, University of Zurich, Winterthurerstrasse 190, 8057 Zürich, Switzerland

**Keywords:** aquatic insects, climate change, land-use, SPEAR_Pesticide_ index, temperature niche, temporal trends

## Abstract

Recently, a plethora of studies reporting insect declines has been published. Even though the common theme is decreasing insect richness, positive trends have also been documented. Here, we analysed nationwide, systematic monitoring data on aquatic insect richness collected at 438 sites in Switzerland from 2010 to 2019. In addition to taxonomic richness, we grouped taxa in accordance with their ecological preferences and functional traits to gain a better understanding of trends and possible underlying mechanisms. We found that in general, richness of aquatic insects remained stable or increased with time. Warm-adapted taxa, common feeding guilds and pesticide-tolerant taxa showed increasing patterns while cold-adapted, rarer feeding guilds and pesticide-sensitive taxa displayed stable trends. Both climate and land-use-related factors were the most important explanatory variables for the patterns of aquatic insect richness. Although our data cover the last decade only, our results suggest that recent developments in insect richness are context-dependent and affect functional groups differently. However, longer investigations and a good understanding of the baseline are important to reveal if the increase in temperature- and pesticide-tolerant species will lead to a decrease in specialized species and a homogenization of biotic communities in the long term.

## 1. Introduction

In recent years, many studies have reported declines in insect richness [1–7]. Nevertheless, it is difficult to generalize trends since regional, taxa- and ecosystem-specific patterns exist [8–13]. Although terrestrial insects appear to be declining in many areas, aquatic insects have more frequently been reported to be increasing [14]. However, studies have to be interpreted with caution as they cover different time periods, regions and study designs [15,16]. Most importantly, factors such as the shifting baseline syndrome [17] and the replacement of sensitive taxa with tolerant ones must be taken into consideration when explaining increasing trends in aquatic insect richness [18].

Even though patterns in insect richness are well-described, the macroecological factors discussed for recent developments in insect richness are manifold [19]. Among the most often cited predictors for the temporal and spatial distribution of insect richness are climate and land-use changes as well as pesticide application, pollution and invasive species [20–24]. Since these factors may act synergistically or may cause opposing effects, it is difficult to generalize their combined impact on insect richness [25–27].

Here, we focus on recent trends in richness of aquatic insects and other macroinvertebrates in Switzerland and their underlying drivers. We analysed nationwide, systematic monitoring data on aquatic insect richness collected at 438 sites from 2010 to 2019. In Switzerland, the availability of spatially representative multi-taxa monitoring data offers a unique opportunity to elucidate potential causes of recent trends in insect richness. We focused both on species richness data of the insect orders Ephemeroptera, Plecoptera and Trichoptera (EPT) and on family richness data of other groups of macroinvertebrates. We expected to see no clear trends in richness patterns due to the rather short time span of 10 years. If at all, we anticipated an increasing richness of warm-adapted taxa at higher altitudes as a response to warming due to climate change. For cold-adapted species, we did expect even smaller differences in richness in this short time period due to a lag in response time for species extinctions [28]. To further explore richness patterns, we grouped taxa according to their functional feeding guilds (FFG) and their sensitivity towards pesticides. The aim was to analyse which groups contribute most to the observed trends and to obtain an indication about potential mechanisms underlying recent patterns in insect richness. Furthermore, we developed a predictive model of aquatic insect richness by both considering factors operating at monitoring sites and at the catchment level.

## 2. Material and methods

### (a) Macroinvertebrate biomonitoring data

We used the presence and absence data of macroinvertebrates collected within the scope of the Swiss Biodiversity Monitoring (BDM) [21–23,29,30]. In this programme, standardized multi-habitat sampling in rivers and streams at 491 sites located along a regular grid across Switzerland is used to inform about the nationwide state and temporal trends of biodiversity [31]. It is based on kick-samples of eight microhabitats per site that are pooled before taxa identification. The data cover 10 years from 2010 to 2019 and include both species-level information on the insect orders EPT (may-, stone- and caddisflies) and family-level information on all EPT and non-EPT macroinvertebrate taxa. For some EPTs, only data for species-complexes were available (electronic supplementary material, table S1) and are subsequently treated as species. For non-EPT macroinvertebrate taxa, a few are treated at a level higher than family (such as Nemathelminthes, Oligochaeta: electronic supplementary material, table S2). Since abundance data were not available, we focused on the metrics species and family richness. As a compromise between temporal sampling completeness and financial constraints, each year, a random subset of one-fifth of all BDM monitoring sites was sampled with equal sampling effort [29] (i.e. each site was resampled after 5 years). Here, we focus on the 438 sites for which EPT and non-EPT were available for both sampling years. We allocated each monitoring site to its respective altitudinal zone [32] and separately considered the colline (*N* = 81) and montane zone (*N* = 205) while we pooled the subalpine and alpine zones (*N* = 152) because of the relatively low number of monitoring sites in the alpine zone (electronic supplementary material, figure S1). In each year, monitoring sites were sampled across altitudinal zones (electronic supplementary material, table S3). Overall, monitoring sites covered elevations from 197 to 2546 m.a.s.l.

### (b) Functional traits/ecological preferences

Information on temperature niches and FFG of macroinvertebrates was obtained from the freshwaterecology.info database [33]. Data on the sensitivity to pesticides according to the SPEAR_Pesticide_ index (based on sensitivity to organic toxicants, generation time, mobility and the presence of aquatic live stages during the pesticide application period) were taken from the database systemecology.de [34,35]. Since knowledge on temperature niches and feeding guilds was available at species level and aggregation of such species-level traits to higher taxonomic units such as family level is problematic [33], we focused our trait analysis on EPT species only. Conversely, for SPEAR_Pesticide_ sensitive and insensitive taxa, information also exists at the family level [36]. As EPT species belong nearly exclusively to the category SPEAR_Pesticide_ sensitive, we decided to analyse sensitivity towards pesticides on the level of macroinvertebrate families only. Further information on temperature niches, pesticide sensitivity and feeding guilds can be found in the electronic supplementary material, S1.

### (c) Environmental predictors

Based on *a priori* knowledge [21–23], we selected ambient air temperature (for a justification of using air temperature instead of water temperature, see electronic supplementary material, figure S2), flow velocity, livestock unit density (LUD) in the catchment, percentage of forest cover and an insecticide land-use index as potential explanatory variables predicting macroinvertebrate richness [23]. While the former two are site-level variables, the latter three describe major environmental conditions at the catchment level. LUD and insecticide application rate (IAR) served as surrogate measures for agricultural intensity, whereas the percentage of forest cover was chosen to reflect the fraction of semi-natural habitats in the catchment devoid of agricultural activity. A more detailed description of the chosen environmental predictors, their calculations as well as frequency plots can be found in the electronic supplementary material, table S4 and figure S3.

### (d) Statistical analysis

All statistical analyses were performed in R v. 4.0.3 [37]. Hereafter, the term species richness is used to refer to local species richness, i.e. the number of taxa present at a monitoring site. The distribution of species richness of EPT species, EPT functional groups, the number of families and SPEAR_Pesticide_ sensitive and insensitive taxa along time was analysed with generalized linear models (GLMs) with a Poisson error distribution. When we detected overdispersion in the data, we used a negative binomial error distribution. For analysing the predictors of macroinvertebrate richness, we conducted multivariate analysis separately for EPT and family richness as well as for EPT functional groups and SPEAR_Pesticide_ sensitive and insensitive taxa as response variables. All environmental predictors were included as fixed effects in the starting models while the monitoring year was added as a random effect. In addition to linear terms, we also included quadratic terms of temperature, percentage of forest, LUD and velocity to account for possible unimodal relationships between these predictors and the response. Prior to analysis, all explanatory variables were standardized by subtracting the mean and division by the standard deviation. A backward selection approach was employed by successively removing variables in case their significance level was *p* > 0.05. We computed models with the *‘glmmTMB’* package [38] and evaluated model fit with fivefold cross-validation (*‘caret’* package [39]). Average marginal effect sizes of predictors were calculated with the *‘mfx’* package [40].

## 3. Results

**Figure 1.**
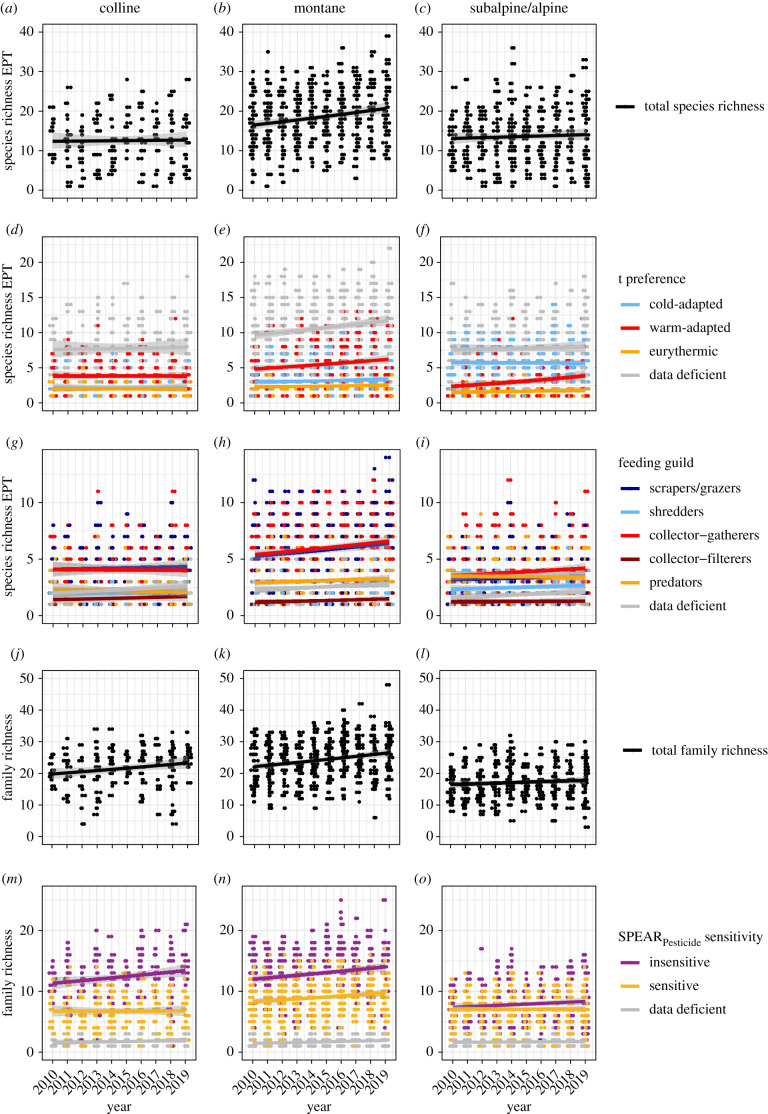
Patterns of EPT species and macroinvertebrate family richness over time in the period from 2010 to 2019. (*a–c*) Species richness of all EPT species in colline (*a*), montane (*b*) and subalpine and alpine (*c*) zones, respectively; (*d–f*) EPT species divided into their temperature niches (cold-adapted: less than 10°C water temperature; warm-adapted: greater than or equal to 10°C; eurythermic: no temperature preference); (*g–i*) EPT species assigned to the FFGs scrapers/grazers, shredders, collector–gatherers, collector–filterers and predators; (*j–l*) number of families; (*m–o*) families divided into SPEAR sensitive and insensitive families. Data deficient denotes taxa with no information available. Dots represent original measurements on monitoring sites. For visibility, data are minimally jittered along the *x*-axis. Trend lines were computed using GLMs. The grey shaded areas around trend lines depict the 95% confidence intervals.

Both EPT and family richness displayed stable or increasing trends in the period from 2010 to 2019, dependent on the altitudinal zones. While EPT richness increased in the montane zone and remained stable in the colline and subalpine/alpine zones (figure 1*a–c*), family richness increased in both the colline and montane zones (figure 1*j–l*). The increasing trends were driven by different groups of taxa (electronic supplementary material, table S5). Warm-adapted EPT species increased over time in both the montane and subalpine/alpine zones while cold-adapted and eurythermic EPT species showed stable trends (figure 1*d–f*). Regarding FFGs, scrapers/grazers and collector–gatherers were the only two groups showing a positive trend, the former in the montane and the latter in both the montane and subalpine/alpine zones (figure 1*g–i*). Richness of SPEAR_Pesticide_ insensitive taxa increased with time in all three altitudinal zones, whereas SPEAR_Pesticide_ sensitive taxa exhibited a positive trend solely in the montane zone (figure 1*m–o*).

**Figure 2.**
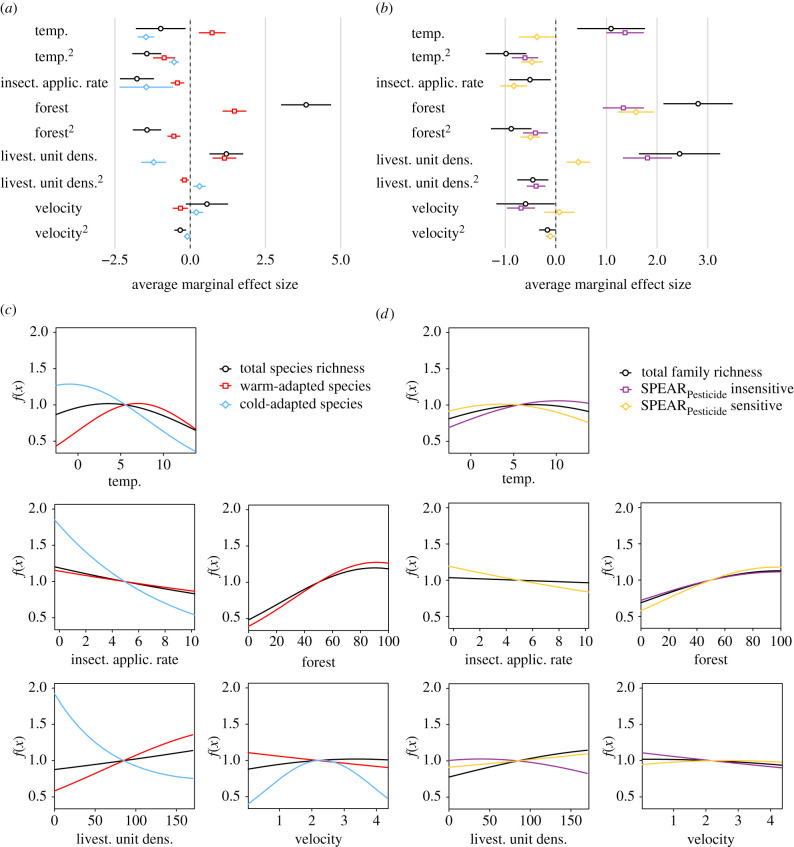
Average marginal effect sizes and predictors for overall EPT species richness, warm- and cold-adapted species (*a,c*), for overall family richness and SPEAR_Pesticide_ sensitive and insensitive families (*b,d*). (*a,c*) Effect sizes for predictors retained in the best model including coefficients and their 95% confidence intervals; (c) predictors retained in the best model for EPT species richness (black line) and warm- (red line) and cold-adapted (blue line) species, (*d*) for family richness (black line) and SPEAR_Pesticide_ insensitive (purple line) and SPEAR_Pesticide_ sensitive families (yellow line). temp. = temperature, insect. applic. rate = insecticide application rate, forest = % forest cover in the catchment, livest. unit dens. = livestock unit density, velocity = flow velocity. *f*(*x*) shows the response curve of the predictor with *f*(*x*) *=* *ax^2^* *+* *bx*, where *a* and *b* are the coefficients for the quadratic and linear terms of the predictor, respectively. Note that *f*(*x*) is a logarithmic function, which was back-transformed to facilitate interpretation.

The strength and direction of predictors for EPT and family richness as well as for different ecological preferences and functional groups differed, which may be indicative of distinctive underlying mechanisms. For both EPT species (figure 2*a*) and family richness (figure 2*b*; electronic supplementary material, figure S4), all five predictors—temperature, IAR, percentage of forest, LUD and velocity—were retained in the best model. The relationship with air temperature was unimodal with an optimum at 5.2°C and 7.2°C, respectively, while the relationship with the other predictors was curvilinear or linear (figure 2*c,d*; electronic supplementary material, figure S5). For warm-adapted species, the optimum temperature was higher than for cold-adapted species (7.02°C versus −0.98°C; figure 2*c*), and warm-adapted species showed a positive relationship with LUD, while it was negative for cold-adapted species (figure 2*c*). The relationship with flow velocity was negative for warm-adapted and unimodal for cold-adapted species (figure 2*c*). Whereas FFGs largely displayed similar trends (electronic supplementary material, figures S4 and S5), patterns for SPEAR_Pesticide_ insensitive and SPAR_Pesticide_ sensitive taxa varied: comparable to warm- and cold-adapted species, SPEAR_Pesticide_ insensitive taxa showed a higher optimum temperature than sensitive taxa (10.22°C versus 3.5°C; figure 2*d*). The amount of explained variance predicted with fivefold cross-validation ranged from *R*^2^ = 0.16 to *R*^2^ = 0.59 (predators and SPEAR_Pesticide_ insensitive taxa, respectively; electronic supplementary material, table S6).

## 4. Discussion

In this study, we analysed recent trends in the richness of aquatic insects in Switzerland and linked them to functional traits and ecological preferences of species. Even though our study covers the period from 2010 to 2019 only, and contrary to our expectations, we found strong signals of stable or increasing trends in insect richness for specific altitudinal zones and groups of taxa.

Warm-adapted species richness increased in the montane and subalpine/alpine zones, which may be a consequence of increases in the average temperature during the investigated time period (electronic supplementary material, figure S6) and indicative of shifting stream macroinvertebrate communities as a result of climate change [41]. We found a small increase in altitude for both warm- and cold-adapted species over the study period (electronic supplementary material, figures S7–S9). While the latter showed stable species richness across altitudinal zones, possibly, this pattern may be a transient state that could lead to the local decline and eventual extinction of cold-adapted species [40]. Ultimately, this may result in the prevalence of more generalist warm-adapted species and decreasing biodiversity at broad spatial scales, provided that interspecific competition and species displacement occurs [41–44].

Primarily generalist feeding groups, such as scrapers and collector–gatherers, increased in richness with time as did mainly families insensitive to pesticides. This finding does not support that improved water quality leads to increasing aquatic insect richness. Rather, it may indicate that common insensitive taxa become even more common in times of climate change, which may result in biotic homogenization [45,46]. Therefore, the present pattern of increasing taxonomic richness has to be evaluated critically. However, we did not find a signal of biotic homogenization as beta diversity remained stable over the study period (electronic supplementary material, figure S10).

Regarding the drivers of aquatic insect richness, all groups displayed unimodal relationships with temperature with the optimum being higher for warm-adapted and SPEAR_Pesticide_ insensitive taxa. The relationship with IAR was consistently negative with the exception of SPEAR_Pesticide_ insensitive taxa, indicating a negative impact of pesticides or other agricultural factors on aquatic insect richness [47]. The percentage of forest in the catchment—an indicator of extensive land-use—exerted positive effects throughout, mirroring the positive influence of riparian forest vegetation on macroinvertebrate richness [48]. Temperature, flow velocity and LUD all influence oxygen saturation, which may be an important driver of aquatic insect richness [49]. Contrasting patterns of LUD for warm- and cold-adapted species could indicate that warm-adapted species are less sensitive towards allochthonous nutrient input than cold-adapted species [50].

The lack of abundance data due to methodological constraints of the semi-quantitative sampling procedure prevented the test of additional hypotheses, such as functional consequences or potential changes in the population size of cold-adapted species due to climate change [41,51]. Furthermore, the fact that non-EPT taxa were only available at family level limited ecological inferences as functional diversity may be high within genera and species turnover may be overlooked [33,52]. Moreover, our model inferences were generally characterized by intermediate levels of explained variance, which is most likely due to the existence of other important predictors or processes that could not be considered in the models (e.g. food availability and biotic interactions) and uncertainty in the included predictors. Most importantly, our study covers data from 10 different years only, the minimum amount required for robust time-series analysis according to the literature, which contributes to the difficulty of predicting future trends [12,53]. Nevertheless, we expect that the presented increase in taxonomic richness will continue under climate change in the coming years.

In conclusion, our analysis suggests that global change—including climate and land-use changes—drives the observed temporal trends of stable or increasing aquatic insect richness in Switzerland during the last decade. However, many major impairments in aquatic ecosystems have taken place already earlier [15,54]. Thus, longer time series and a good understanding of the baseline are paramount to reveal whether the observed trends in taxonomic richness are stable and whether the increase in temperature- and pesticide-tolerant species will lead to replacements of specialized species and a functional homogenization of biotic communities in the future.

## Data Availability

All data are provided in the electronic supplementary material [55].
